# Genome-Wide Analysis and Abiotic Stress-Responsive Patterns of *COBRA-like* Gene Family in *Liriodendron chinense*

**DOI:** 10.3390/plants12081616

**Published:** 2023-04-11

**Authors:** Chen Qiu, Jinhui Chen, Weihuang Wu, Bojun Liao, Xueyan Zheng, Yong Li, Jing Huang, Jisen Shi, Zhaodong Hao

**Affiliations:** 1 State Key Laboratory of Tree Genetics and Breeding, Co-Innovation Center for Sustainable Forestry in Southern China, Nanjing Forestry University, Nanjing 210037, China; 2 National Germplasm Bank of Chinese Fir at Fujian Yangkou Forest Farm, Nanping 353211, China; 3 Jinling Institute of Technology, Nanjing 211169, China; 4 Key Laboratory of Forest Genetics and Biotechnology of Ministry of Education, Nanjing Forestry University, Nanjing 210037, China

**Keywords:** *COBL*, genome-wide analysis, gene expression, qRT-PCR

## Abstract

The COBRA gene encodes a plant-specific glycosylphosphatidylinositol (GPI)-anchored protein (GAP), which plays an important role in cell wall cellulose deposition. In this study, a total of 7 *COBRA-like* (*COBL*) genes were identified in the genome of the rare and endangered woody plant *Liriodendron chinense* (*L. chinense*). Phylogenetic analysis showed that these *LcCOBL* genes can be divided into two subfamilies, i.e., SF I and II. In the conserved motif analysis of two subfamilies, SF I contained 10 predicted motifs, while SF II contained 4–6 motifs. The tissue-specific expression patterns showed that *LcCOBL5* was highly expressed in the phloem and xylem, indicating its potential role in cellulose biosynthesis. In addition, the cis-element analysis and abiotic stress transcriptomes showed that three *LcCOBLs*, *LcCOBL3*, *LcCOBL4* and *LcCOBL5*, transcriptionally responded to abiotic stresses, including cold, drought and heat stress. In particular, the quantitative reverse-transcription PCR (qRT-PCR) analysis further confirmed that the *LcCOBL3* gene was significantly upregulated in response to cold stress and peaked at 24–48 h, hinting at its potential role in the mechanism of cold resistance in *L. chinense*. Moreover, GFP-fused LcCOBL2, LcCOBL4 and LcCOBL5 were found to be localized in the cytomembrane. In summary, we expect these results to be beneficial for research on both the functions of *LcCOBL* genes and resistance breeding in *L. chinense*.

## 1. Introduction

Cell walls play a vital role in protecting plants against biotic and abiotic stresses, as well as in plant support and protection [1,2,3]. The primary cell wall, consisting mainly of cellulose, hemicellulose, pectin and proteins, surrounds each plant cell. The secondary cell wall consists mainly of cellulose, hemicellulose and lignin, and, in specific cell types, is deposited between the plasma membrane and the primary cell wall after cell enlargement stops [4]. Cellulose, which is the most abundant biopolymer on Earth, is accumulated in plant cell walls [5]. Cellulose is a linear homopolysaccharide that makes up long and rigid microfibrils and is the load-bearing structure in cell walls [6]. The organization of cellulose is critical for directed plant growth [7,8]. The biosynthesis of cellulose is mediated by cellulose synthase (CesAs), which forms a rosette-like cellulose synthase complex (CSC) on the plasma membrane [5]. A genome-wide association study (GWAS) for culm cellulose content in barley (*Hordeum vulgare*) identified *HvCOBRA*, associated with cellulose synthesis [9]. The factors involved in cellulose synthesis were identified by Affymetrix microarray analysis in *Arabidopsis thaliana*, which indicated that *COBRA* homologous gene *COBL4* was among the top 10 genes co-expressed with *CESA4*, *7* and *8*, indicating a potential role of *COBRA* gene in protocell wall cellulose deposition [10].

COBRA is an important glycosylphosphatidylinositol (GP-I) anchor protein (GAP) that is mainly distributed along the longitudinal sides of the rapid elongation region of root cells and is involved in cellulose deposition to regulate cell expansion [11]. The COBRA protein contains four conserved domains: the N-terminal protein-targeting domain, the carbohydrate-binding motif domain, the conserved CCVS domain and the hydrophobic C-terminal domain [12]. Both the N-terminal domain and the hydrophobic C-terminal domain are cleaved after being translated [13]. The protein contains a GPI-anchored ω site in its C-terminal signal peptide, and the ω residue is cleaved and then attached to the C-terminus of the protein [14]. The N-terminal CBM domain preferentially interacts with crystalline cellulose. The C-terminus is where part of the GPI is located, and GPI anchoring is a post-translational modification that promotes the transport of certain proteins to the cell wall [15]. The CCVS domain is the third conserved domain in COB and is thought to be involved in disulfide bond formation or metal ion binding [16].

At present, *COBL* gene family members have been identified in *A. thaliana* (12) [14], *Populus trichocarpa* (18) [17], *Oryza sativa* (10) [18], *Zea mays* (9) [19], *Pyrus bretschneideri* (16) [20] and other plants. Initially, the *COBL* family was identified in *Arabidopsis*, having been isolated in a screening of *Arabidopsis* seedlings with abnormally extended roots [21]. Subsequently, functional studies were carried out in *O. sativa* [14], *Zea mays* [22], *Triticum aestivum* [23] and other monocotyledon plants. The results showed that *COBL* genes regulate the growth and development of plants. In *Sorghum bicolor*, an *SbBC1* single-nucleotide mutation resulted in a decrease in mechanical strength, a decrease in cellulose content and an increase in lignin content. *SbBC1* may mediate the mechanisms of cellulose biosynthesis and cell wall remodeling [24]. In wheat, the *TaCOBL* gene regulates seed coat and seed development by participating in the cellulose biosynthetic process of the plant cell wall and seed coat development [23]. In tomatoes, the *SlCOBRA-like* gene plays an important role in the epidermal cell walls of the fruits, increasing the hardness of the fruits and extending their shelf life [25]. *COBLs* also play important roles in adapting to stress. Genes related to drought, salinity and jasmonic acid (JA) stress were found in wheat [23], upland rice [26], *Saccharum spontaneum* [27] and *Arabidopsis* [28].

*L. chinense* belongs to the magnolia family, an ancient relict plant that plays an important role in evolution [29]. Currently, there are only two species in this genus, namely, *L. chinense* and *Liriodendron tulipifera* [30]. *Liriodendron hybrids* is a hybrid of *L. chinense* and *L. tulipifera*, with hybrid advantages that make it superior to its parent species in terms of flower color, timber properties and growth amount, and it is an important ornamental and economic tree species in China [31]. The genome sequencing and analysis of *L. chinense* showed that Magnoliaceae appeared before the differentiation of eudicots and monocots, which provided new ideas on the evolutionary status of Magnoliaceae and important support for the study of the molecular mechanisms related to *L. chinense* [30].

Based on previous reports, the *COBL* gene is involved in the cell wall biosynthesis of plant roots, stems, leaves and other mechanical tissues and plays a role in root development and adaptation to environmental stresses. Therefore, understanding the biological function of the *COBL* gene is of great significance for studying the mechanical strength and stress of plant stems. Currently, no studies on the identification of the *COBL* genes in *Liriodendron* have been reported. In this study, the genome data of *L. chinense* were analyzed to identify the members of the *COBL* gene family at the whole-genome level, and the evolutionary relationships, conserved motifs and chromosomal localizations of the members of the *COBL* gene family were analyzed using bioinformatics. The transcriptome data in different *organs* and under cold, heat and drought stress were analyzed to identify the gene expression pattern of *COBL* family members. Finally, quantitative reverse-transcription PCR (qRT-PCR) analysis further confirmed the expression levels of *LcCOBL* genes in multiple organs and under cold abiotic stresses. A subcellular localization experiment was conducted to verify the expression location of LcCOBL proteins. These results will provide a valuable foundation for further functional studies of *LcCOBL* genes in cellulose biosynthesis and under abiotic stresses.

## 2. Results

### 2.1. Identification of the COBL Gene Family in the L. chinense Genome

Seven COBL proteins were identified in *L. chinense* with BLAST using Arabidopsis COBL amino acid sequences as reference. They were submitted online to the NCBI-CDD website for conserved domain verification, and, finally, seven *L. chinense* COBL proteins were identified. According to their positions on the chromosome, they were named LcCOBL 1–7. The lengths of the 7 reported LcCOBL proteins to range from 230 to 655 aa, and their molecular weights range from 25.30 to 73.66 kDa (Table 1). The hydrophilicity values of all the LcCOBL proteins are less than 0 (Table 1), indicating that they are hydrophilic proteins (Figure S1). However, LcCOBL2, 4 and 5 in the LcCOBL family lack a GPI modification site (Figure S2). The signal peptide prediction results of LcCOBL proteins showed that all LcCOBL proteins had N-terminal signaling peptides (Figure S3). TMHMM results showed that only LcCOBL2 had 1 transmembrane domain (Figure S4). The subcellular localization predictions showed that all the LcCOBLs were located on the cell membrane, while LcCOBL2 may also play a role in chloroplasts (Table 1).

### 2.2. Phylogenetic Analysis of LcCOBL Proteins

**Figure 1:**
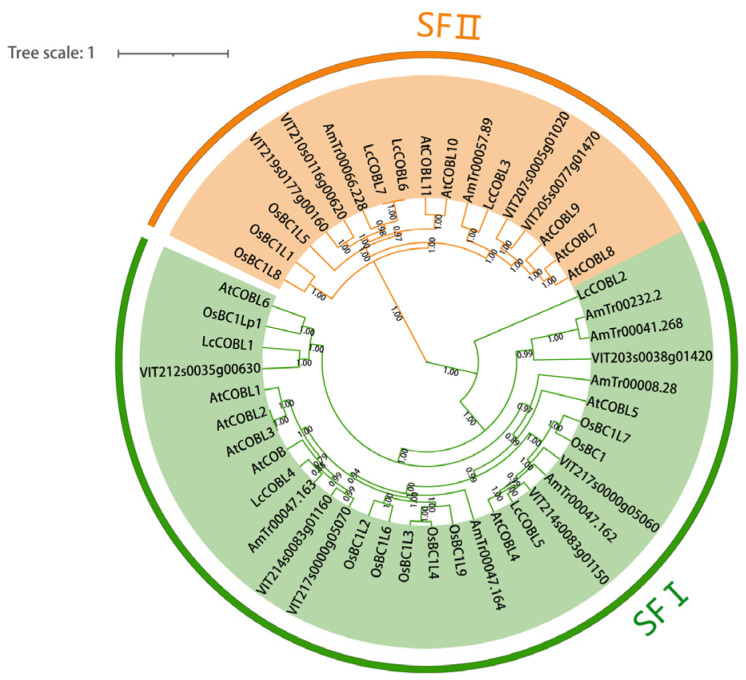
Phylogenetic tree of COBL protein family in *L. chinense*, *A. thaliana*, *O. sativa*, *V. vinifera* and *A. trichopoda*. The full-length COBL protein sequences were aligned using the clustalx tool, and the Bayesian method was used to construct the phylogenetic tree in BEAUti. Finally, the tree was visualized with the online iToL. The bootstrap values are supported by 1000 replications and are shown beside the branches. According to the phylogenetic tree, the seven LcCOBLs could be divided into two subfamilies (SF I and SF II), where SF I stands for LcCOBL subfamily I and SF II stands for LcCOBL subfamily II.

In order to explore the phylogenetic relationships between the COBL proteins, we constructed a phylogenetic tree using 7, 12, 11, 11 and 8 COBL proteins from *L. chinense*, *A. thaliana*, *O. sativa*, *Vitis vinifera* and *Amborella trichopoda*, respectively (Figure 1). The results showed that seven LcCOBLs could be divided into two subfamilies, i.e., SF I and SF II, based on the phylogenetic tree (Figure 1). Four LcCOBLs were grouped in SF I, including LcCOBL1, LcCOBL2, LcCOBL4 and LcCOBL5, while the remaining three LcCOBLs were grouped in SF II. As for *A. thaliana*, *O. sativa*, *V. vinifera* and *A. trichopoda*, SF I contained seven, eight, seven and six family members, while SF II contained five, three, four and two family members, respectively. The phylogenetic grouping of the LcCOBL proteins was further confirmed with a phylogenetic tree constructed using only LcCOBL proteins (Figure S2).

### 2.3. Analyses of Locations, Structures and Conserved Motifs of LcCOBL Genes

**Figure 2:**
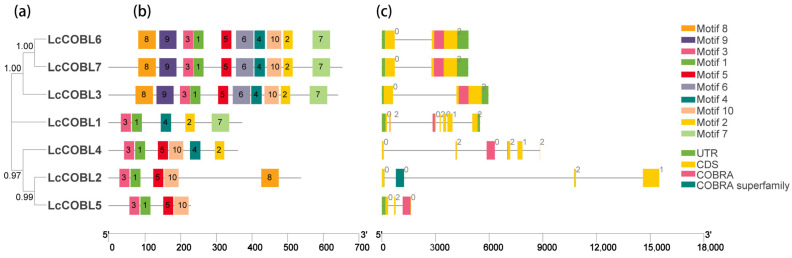
Conserved motifs and structures of *LcCOBL* genes. (**a**) ClustalW was used for multi-sequence alignment of LcCOBL protein sequences. A maximum likelihood (ML) tree was built using MEGA X and 1000 bootstrap repeats. (**b**) The conserved motifs of LcCOBL proteins were revealed using MEME analysis. The colored box on the right represents 10 motifs. (**c**) The gene structures. Yellow boxes, black lines, green boxes, pink boxes and dark green boxes represent exons, introns, UTRs (untranslated regions), COBRA domains and COBRA superfamily domains, respectively.

**Figure 3:**
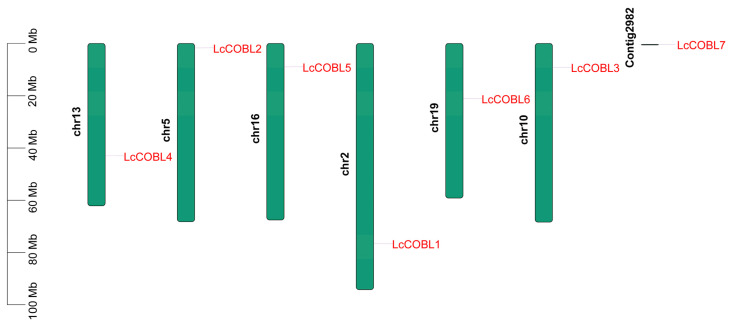
Chromosomal location analysis of *LcCOBLs*. The size of a chromosome is expressed by its relative length. Scale bar on the left indicates the chromosome lengths (Mb).

In order to study the structural characteristics of the *LcCOBL* genes, we analyzed the conserved motifs and the exon–intron structures of the seven *LcCOBL* genes (Figure 2). Among these 10 motifs, only motifs 1 and 3 were possessed by all the LcCOBL proteins. Additionally, motif 4 contained a CCVS-conserved motif of the COBRA protein (Figure S3). The motif differences between the subfamilies were greater than those within the same subfamily, suggesting functional conservation among LcCOBL proteins within the same subfamily (Figure 2a,b). The *LcCOBL* genes contained 2–7 exons, and most of the evolutionarily related exon–introns shared similar structures (Figure 2c). In addition, the analysis of the conserved domains of the LcCOBLs showed that all the LcCOBLs possessed a COBRA domain or a COBRA superfamily domain (Figure 2c). These results further validate the reliability of the identified *LcCOBL* gene family and shed light on its functional evolution. The chromosomal distributions of the *LcCOBL* genes were determined based on the genome-wide data of *L. chinense*. The chromosomal localization analysis showed that seven *LcCOBL* genes were uniformly located on six chromosomes and one contig of *L. chinense* (Figure 3). There was only one gene on each chromosome with no tandem gene replication event.

### 2.4. Cis-Element Analysis of LcCOBL Promoters

**Figure 4:**
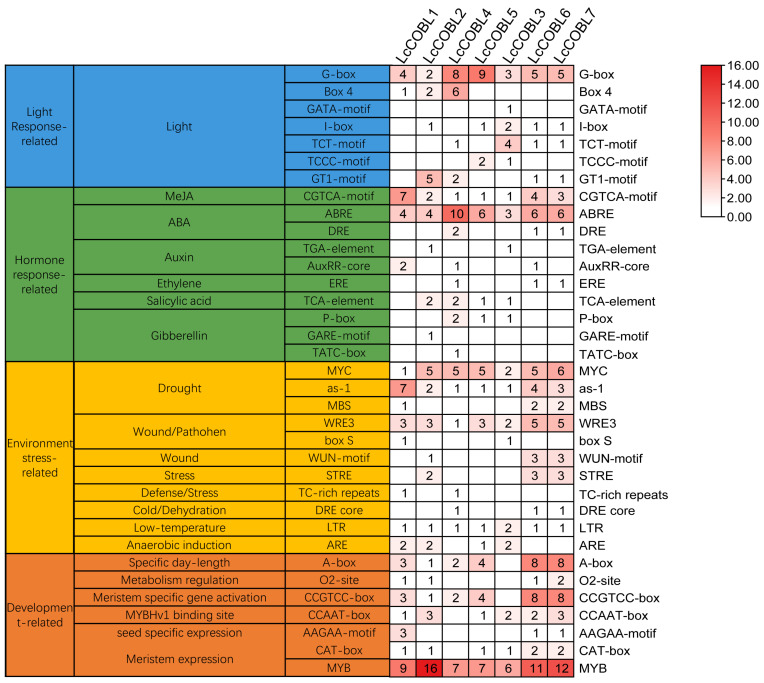
Analysis of cis-elements on *LcCOBL* promoters. Cis-elements in the upstream 2000 bp area of *LcCOBL* promoters. Different colors in the table indicate different types of promoters, and numbers indicate the numbers of different promoter elements.

Cis-elements distributed on gene promoters provide targets that bind to transcription factors, whereby gene expression patterns are activated or inhibited in the processes of plant growth and development and coping with external environmental stresses. We predicted the promoter cis-acting elements through the PlantCARE, which is based on probabilistic sequence models (e.g., Gibbs Sampling) [32]. Based on the conservation of the promoter sequences [33], motifs that have been determined in other species were selected from the screened elements for possible functional analysis in *L. chinense*. We found four major types of cis-acting elements i.e., related to light, hormones, environmental stress or developmental responsiveness, distributed on the promoter regions of the *LcCOBLs* (Figure 4). Among the light-response-related cis-elements, G-box was the most abundant type, which can be found within all *LcCOBL* promoters. Regarding hormones and environmental stress, we found that the ABA- (ABRE) and drought-related (MYC and as-1) cis-elements were the most abundant cis-elements, showing that *LcCOBLs* might play an important role in the response to drought stress. As for the development-related cis-elements, we found that the most abundant type has a function in meristem expression.

### 2.5. Organspecific Expression Patterns of LcCOBLs

**Figure 5:**
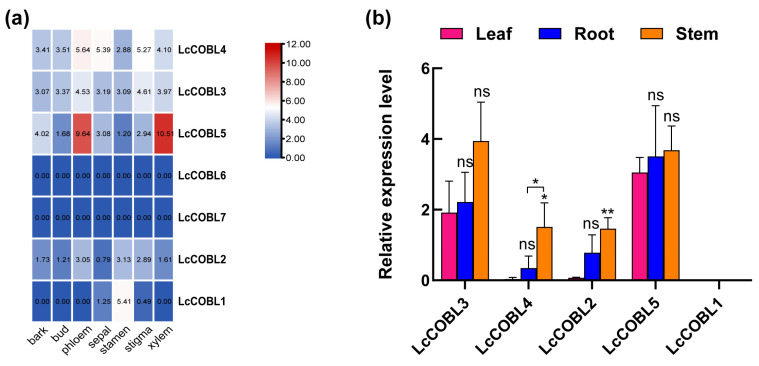
The expression patterns of *LcCOBL* genes in different *L. chinense* organs. (**a**). Expression profiles of *LcCOBL* genes in bark, bud, phloem, sepal, stamen, stigma and xylem tissues. The heatmap data were averaged and plotted using TBtools, with red representing a high expression level and blue representing a low expression level. (**b**) qRT-PCR analysis of *LcCOBL* genes in different organs including leaves, roots and stems. The expression levels of related genes were calculated with 2^−ΔΔCt^ using a leaf as control. Mean values ± SE are shown for the 3 replicates, and the levels of significance relative to the control are ns: no significant difference, * *p* < 0.05 and ** *p* < 0.01.

To further explore the potential functions of the *LcCOBL* family in tissue development, we investigated the expression patterns of the *LcCOBLs* across different organs of *L. chinense* (Figure 5a). According to the transcriptome data regarding eight tissues, namely bark, bud, phloem, sepal, stamen, stigma and xylem. We found that five *LcCOBL* genes were expressed in these selected organs, except for two genes (*LcCOBL6* and *LcCOBL7*). Moreover, these five *LcCOBL* genes were mainly expressed in the phloem, stamen, stigma and xylem tissues, with almost no expression in the bark, bud or sepal tissues. Interestingly, *LcCOBL5* was mainly expressed in the phloem and xylem tissues, showing its potential role in stem growth. Furthermore, a quantitative reverse-transcription PCR (qRT-PCR) analysis confirmed the higher expression of *LcCOBL5* in the stems than in the roots and leaves (Figure 5b).

### 2.6. Expression Patterns of LcCOBLs in Response to Abiotic Stresses

**Figure 6:**
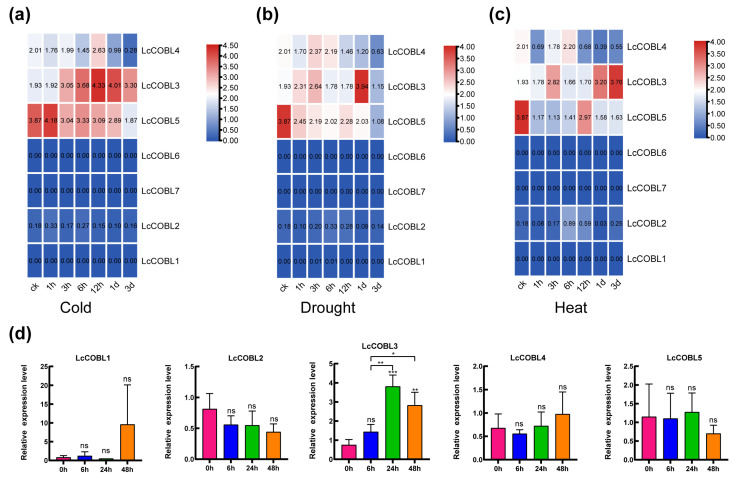
The expression patterns of *LcCOBL* genes in *L. chinense* under abiotic stresses. (**a**–**c**) Expression profiles of *LcCOBL* genes in *L. chinense* under biotic stresses, including cold (**a**), drought (**b**) and heat (**c**) stress. The heatmap data were averaged and plotted using TBtools, with red representing a high expression level and blue representing a low expression level; (**d**) qRT-PCR analysis of *LcCOBL* genes under cold abiotic stress. The expression levels of related genes were calculated with 2^−∆∆Ct^ using 0 h as control. Mean values ± SE are shown for the 3 replicates, and the levels of significance relative to the control are ns: no significant difference, *: 0.01 ≤ *p* < 0.05, **: 0.001 ≤ *p* < 0.01 and ***: *p* < 0.001.

To analyze the responses of the *LcCOBL* genes to abiotic stresses, we examined the expression levels of these *LcCOBLs* under cold, drought and heat stress based on the available RNA-seq data (Figure 6a–c). The results show that three *LcCOBLs*, i.e., *LcCOBL3*, *LcCOBL4* and *LcCOBL5*, transcriptionally responded to these abiotic stresses. Specifically, *LcCOBL3* was highly up-regulated in response to these abiotic stresses and peaked at 12–24 h, 1 d and 3 d under cold, drought and heat stress, respectively. In contrast, *LcCOBL5* was dramatically down-regulated as soon as it was exposed to these stresses. Compared with *LcCOBL5* and *LcCOBL3*, the expression dynamic of *LcCOBL4* was less violent, although it first increased and then decreased.

To further determine whether the expression levels of the *LcCOBL* genes were influenced by abiotic stresses, *Liriodendron* seedlings treated with low-temperature conditions (4 °C) were collected to quantify the expression levels of the *LcCOBLs* using qRT-PCR analysis. The results show that each gene has a different expression pattern after cold treatment (Figure 6d). The expression level of *LcCOBL3* in the experimental group was significantly increased compared with the control group, while the expression level of *LcCOBL2* in the treatment group was decreased. *LcCOBL4* and *LcCOBL5* showed opposing expression patterns. *LcCOBL4* expression was downregulated at 6 h and then upregulated. *LcCOBL5* was upregulated at 24 h and then downregulated. This suggests that the *LcCOBLs* exhibit different expression trends under stress treatment, indicating that these genes respond to the regulation of abiotic stress to varying degrees.

### 2.7. Subcellular Localization of LcCOBL Proteins

**Figure 7:**
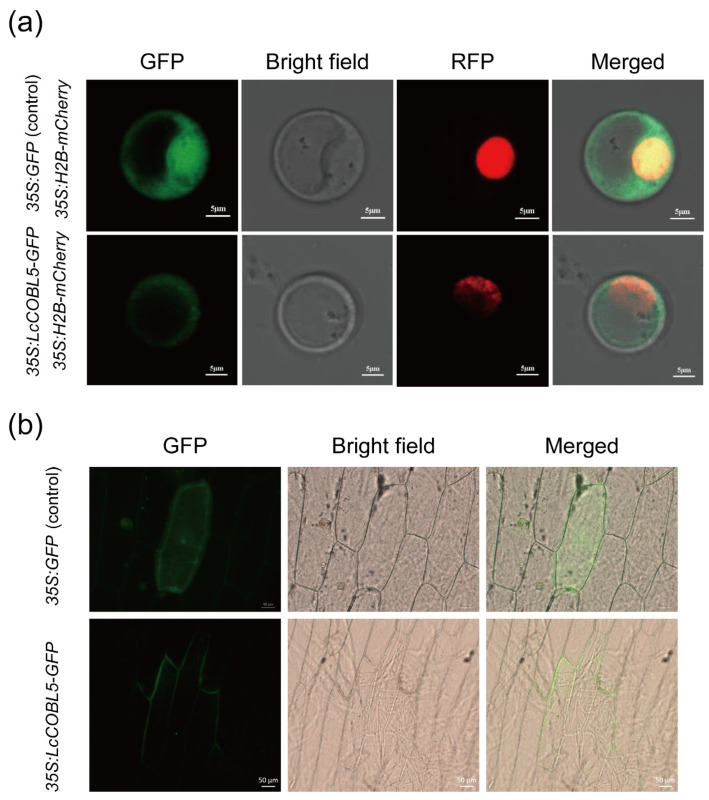
Subcellular localization of LcCOBL5 protein in *L. chinense* calli (**a**) and onion epidermal cells (**b**). In these pictures, GFP indicates green fluorescence photography, bright field indicates bright-field photography, RFP indicates red fluorescence photography and merged indicates the fusion of green fluorescence, red fluorescence and bright field photography. The red fluorescence locates the nucleus, and the green fluorescence locates the LcCOBL proteins.

To further explore the potential roles of these LcCOBL proteins, we cloned the full-length CDSs of three LcCOBLs, i.e., LcCOBL2, LcCOBL4 and LcCOBL5 (Supplementary Table S2). Then, these *LcCOBL* genes without terminating codons were inserted into pJIT166 vectors to obtain *LcCOBL*-*GFP* fusion vectors, which were driven by a 35S promoter. Then, these three vectors were separately transferred into the protoplasts of *L. chinense* calli via PEG-mediated protoplast transformation and transferred into onion epidermal cells by gene gun. At the same time, the *35S:H2B-mCherry* vector was transferred into callus protoplasts as the control for the nuclear localization. The results show that the protoplasts transferred with the *35S:GFP* vector expressed the GFP signal in the whole cell, which was a constitutive expression, while in *35S:H2B-mCherry* lines, the mCherry signal was only observed in the nucleus. Furthermore, for the *LcCOBL5*-*GFP* fusion vectors, the GFP signal was found in the cytomembrane, and the weak fluorescence signal was also observed in the cytoplasm (Figure 7). However, we cannot determine the expression of LcCOBL2, 4 proteins in other organelles except cytomembrane (Figure S7). This requires further experiments to verify.

## 3. Discussion

### 3.1. Functions of LcCOBLs in Stem Development and Stress Responses

In phylogenetic trees, genes with similar clusters may have similar functions. Therefore, LcCOBLs are likely to have similar biological functions to COBL proteins known to be in other species in this group. It was stated that genes with less intron number may be expressed faster than other genes by upstream signals [34]. Combining the gene expression patterns of members of the *COBL* gene families helps to predict the gene functions of the *LcCOBL* gene family members. At present, many genes related to brittle traits, including *OsBCIL4* and *OsBC1*, that have been resolved have been reported in rice, indicating that the *bc* mutant gene in rice controls the culm mechanical strength mainly by influencing cellulose metabolic enzymes [14,35]. A phylogenetic analysis showed that *LcCOBL1* and *LcCOBL3* are homologous with *OsBC1Lp1*, *OsBC1L1* and *OsBC1L8* in rice, respectively, and may be involved in the metabolic synthesis of cellulose. Studies on *Atcob-1* have shown that *AtCOB* is an important factor in the highly anisotropic expansion of plant morphogenesis by participating in the directional growth of cellulose microfibrils [36]. *LcCOBL4* has high homology with *AtCOB* and may be involved in the directional expansion of cellulose microfibrils.

The expression patterns of the *LcCOBLs* in different tissues showed that all *LcCOBLs* except *LcCOBL1* were highly expressed in stems, followed by roots. In the root, stem and leaf tissues of the plant, the cellulose contents in the stems, roots and leaves ranged from the highest to lowest, respectively, which further proves that *LcCOBLs* are associated with cellulose biosynthesis. In *Arabidopsis*, COBL proteins are key regulators of the direction of cell expansion in roots. In the identification of the expression sequence markers of potential homologous genes in other plants, it has been shown that *COBL*-gene-related functions may be necessary for all vascular plants [16]. The content of cellulose in the root tips of *Arabidopsis* mutants was significantly reduced, and the root cells were laterally expanded, suggesting that the regulation of cell directional expansion by *COB* is related to cellulose deposition [11]. In rapeseed (*Brassica napus*), RNA-seq analysis showed that *BnaCOBL9*, *BnaCOBL35* and *BnaCOBL41* were highly expressed in stems with high breaking resistance and may be involved in the stem development and stem-breaking resistance of rapeseed [37]. Combining these findings with those of previous studies, *LcCOBL* genes may be involved in plant cellulose synthesis and enhance stem fracture resistance.

To investigate the functions of the *LcCOBLs* in cold stress, we used qRT-PCR to analyze the expression patterns of the *LcCOBLs* in stems under low-temperature (4 °C) treatment. Over different processing times, different *LcCOBLs* showed different expression trends. Moreover, in the cis-elements analysis of *LcCOBL* promoters, each *LcCOBL* gene had elements related to low temperature. These results suggest that these *LcCOBL* genes may help improve the cold tolerance of plants. Similarly, previous studies have shown that *COBL* genes play important roles in drought and salt tolerance in other species [19,26,27].

### 3.2. Subcellular Localization of LcCOBLs and Their Associations with Potential Functions

Plant extracellular pH is acidic (pH 5.7), and many physiological and external environmental factors can cause changes in extracellular pH [38]. It was found that GFP was pH sensitive and its fluorescence decreased significantly under acidic conditions [39]. Although the shape of the spectrum does not change significantly with pH, the intensity gradually decreases with lowered pH, decreasing to 50% of maximal intensity at a pH of ~6 [40,41]. TMHMM results showed that only LcCOBL2 had 1 transmembrane domain. However, a glycosylphosphatidylinositol (GPI) anchor is an alternative means of attaching a protein to the membrane [42]. As LcCOBL proteins are anchored to the outer layer of the cytoplasmic membrane and in an acidic environment, the GFP signal is weakened, resulting in an unclear fluorescence signal in the result images. The current experimental results show that, for the LcCOBL5-GFP fusion vector, the GFP signal was found in the cytomembrane, the weak fluorescence signal was also observed in the cytoplasm (Figure 7). However, we cannot determine the expression of LcCOBL2, 4 proteins in other organelles except cytomembrane (Figure S7). This requires further experiments to verify.

The result of subcellular localization indicated that the *LcCOBL5* in *L. chinense* calli and onion epidermal cells are localized in the cytomembrane and cytoplasm, which is consistent with the results of other species. By imaging living cells in *Arabidopsis*, it was found that *COBL* was located in particles in the plasma membrane [1,43]. In onion epidermal cells bombarded with an *Ubi::OsBC1L4:GFP* vector, GFP signals were found in the cell wall and plasma membrane [35]. This is consistent with the results of *COB* being observed in the elongation region of *Arabidopsis* roots using transmission electron microscopy. *COB* exists in the plasma membrane and some cell walls [36]. However, in *Cunninghamia lanceolata*, the fused ClCOBL1-RFP protein is transformed into tobacco cells. RFP signals mainly exist in the cell wall and membrane, but there is also a weak signal in the cytoplasm [43]. The COBRA gene encodes a plant-specific glycosylphosphatidylinositol (GPI)-anchored protein (GAP). GPI biosynthesis and transfer to proteins are carried out on the endoplasmic reticulum [13,44]. It may be that the process of GPI biosynthesis and transfer leads to the detection of fluorescence signals in the cytoplasm. Based on the above analysis, there are differences in the expression positions of *COBL* genes in different species, but most of the members are mainly expressed in the plasma membrane, a few in the cytoplasm, indicating that *LcCOBLs* participate in a variety of biological regulation processes.

## 4. Materials and Methods

### 4.1. Identification of the LcCOBLs Family in the L. chinense Genome

*L. chinense* protein sequences were downloaded from (https://hardwoodgenomics.org/Genome-assembly/2630420) (accessed on 3 March 2022). Firstly, the protein sequences of each member of the *Arabidopsis COBL* gene family were downloaded from the *Arabidopsis* genome database (Phytozome) [45] (https://phytozome-next.jgi.doe.gov) (accessed on 3 March 2022). Based on a BLASTp search [46] (E-value ≤ 1 × 10^−5^), *LcCOBL* family members were identified. Second, the HMM file corresponding to the COBRA domain (PF04833) was downloaded from the Pfam protein family database [47,48] (https://www.ebi.ac.uk/interpro/) (accessed on 2 March 2022). The search in the *L. chinense* protein database was carried out via an HMM search, and the sequences containing this domain were screened, and duplicate sequences were removed. Then, the NCBI-CDD database (https://www.ncbi.nlm.nih.gov/Structure/cdd/wrpsb.cgi) (accessed on 3 March 2022) was used to analyze the encoding protein characteristics of the LcCOBLs to ensure that the LcCOBLs had COBRA domains. Finally, seven *LcCOBL* genes were identified in the *L. chinense* genome. Furthermore, the basic features of the LcCOBL proteins of *L. chinense* (sequence length; MW; pI) were identified using ExPasy (https://prosite.expasy.org) (accessed on 30 October 2022). TMHMM Server v. 2.0 (https://dtu.biolib.com/DeepTMHMM) (accessed on 30 October 2022) was used to predict the protein transmembrane domains. The N-terminal signal peptide prediction of LcCOBL proteins was carried out using SignalP-6.0 (https://services.healthtech.dtu.dk/services/SignalP-6.0/) (accessed on 3 March 2023). Target P (https://services.healthtech.dtu.dk/services/NetGPI-1.1/) (accessed on 3 March 2023) was used to analyze the anchor sites at the hydrophobic end of the C-terminal. Subcellular localization prediction was performed using Cell-PLoc 2.0 (http://www.csbio.sjtu.edu.cn/bioinf/Cell-PLoc-2/) (accessed on 30 October 2022).

### 4.2. Phylogenetic Analysis and Classification of the LcCOBL Family

According to the classification of the COBL proteins of Arabidopsis, 7 COBL proteins of *L. chinense* were divided into 2 groups. The COBL family protein sequences of *A. thaliana*, *O. sativa*, *V. vinifera* and *A. trichopoda* were downloaded from the Phytozome database (https://phytozome-next.jgi.doe.gov/) (accessed on 28 March 2022). The full-length COBL protein sequences were aligned using the clustalx tool. TrimAl (‘automated1’ mode) was used to trim the aligned sequences to generate the trimmed MSA file, which was then used to build the COBL phylogenetic tree. Using BEAUti software, the clipped FASTA file was output into an XML format. After the BEAUti program was completed, the TreeAnnotator program was used to construct the Bayesian phylogenetic tree. The TreeAnnotator program set the posterior probability limit to 1.0, the Burnin percentage to 90, the target tree type to maximum branch confidence tree, and the node height to common ancestor heights. Finally, the tree was visualized with the online iToL (https://itol.embl.de/) (accessed on 1 March 2023).

### 4.3. Analyses of the Chromosomal Locations, Structures and Conserved Motifs of LcCOBL Genes

The phylogenetic relationships between the *LcCOBL* genes were analyzed with MEGA X using the maximum likelihood (ML) method with 1000 bootstrap replicates. The position information of the *LcCOBL* genes on the chromosome was picked up from the *L. chinense* annotations using TBtools [49]. MEME (https://meme-suite.org/meme/doc/meme.html) (accessed on 31 March 2022) was further carried out to investigate their conserved motifs [50]. The GFF annotation files and the conserved structure sequences were imported into TBtools to visualize the exon–intron structures and COBRA domains. The GFF annotated files were obtained from the *L. chinense* genome file, and the conservative structural sequences were obtained from the NCBI (https://www.ncbi.nlm.nih.gov/Structure/cdd/wrpsb.cgi) (accessed on 30 October 2022). In addition, the software PlantCARE (https://bioinformatics.psb.ugent.be/webtools/plantcare/html/) (accessed on 7 April 2022) was used to predict the cis-acting elements of the 7 *LcCOBL* genes in the upstream 2000 bp range.

### 4.4. Plant Materials, RNA Extraction, qRT-PCR Analysis, and Transcriptome Sequencing Analysis of Abiotic Stress in L. chinense

The *Liriodendron hybrids* plants used in this study were obtained from the Key Laboratory of Forest Genetics and Biotechnology of the Ministry of Education at Nanjing Forestry University. *Liriodendron hybrids* seedlings generated via somatic embryogenesis were cultured in a greenhouse under light 16 h/dark 8 h conditions (temperature, 25 °C; humidity, 70%).

In the tissue-specific expression experiments, leaf, root and stem tissues were taken from 3-month-old tissue culture bottle seedlings. Under abiotic stress, 3-month-old tissue culture bottle seedlings were cultured in a cold (4 °C) growth chamber. Stem tissues from the treatment group and control group were collected at 0, 6, 24 and 48 h after the experiment and stored at −80 °C.

Total RNA was isolated from each sample using the Eastep^®^Super Total RNA Extraction Kit (Promega), and first-strand cDNA was synthesized from the proposed RNA using a PrimeScript RT Master Mix (Takare). A qPCR SYBR Green Master Mix (Vazyme) was used for real-time quantitative PCR, and the *GAPDH* gene was used as the internal control gene [51]. The real-time PCR cycling parameters were 95 °C for 30 s, followed by 45 cycles at 95 °C for 5 s and 60 °C for 30 s, with a melting curve analysis. All reactions were performed in triplicate to ensure the repeatability of the results. Gene expression levels were calculated using 2^−∆∆Ct^.

The seedlings generated through somatic embryogenesis grown at 22 °C, light 16 h/dark 8 h, 75% relative humidity were treated at 4 °C, 40 °C or 15% PEG 6000 for 1 h, 3 h, 6 h, 12 h, 1 day and 3 days, respectively, and were treated with cold, heat and drought. Each treatment consisted of five replicates for each sampling time, taking leaf tissue samples for RNA-seq analysis. The transcriptome data about cold and heat stress (PRJNA679089) and drought stress (PRJNA679101) could be downloaded from NCBI [52]. The transcriptome data are in regards to eight organs from the author of this paper [30].

### 4.5. Subcellular Localization

In order to verify the prediction results of the subcellular localization of the LcCOBL proteins, we cloned the full-length CDSs of three LcCOBLs, i.e., LcCOBL2, LcCOBL4 and LcCOBL5. Then, these *LcCOBL* genes without terminating codons were inserted into pJIT166 vectors to obtain *LcCOBL*-*GFP* fusion vectors, which were driven by a 35S promoter. Then, these three vectors were separately transferred into the protoplasts of *L. chinense* calli via PEG-mediated protoplast transformation and transferred into onion epidermal cells by gene gun. At the same time, the *35S:H2B-mCherry* vector was transferred into callus protoplasts as the control for the nuclear localization. The plasmids of the corresponding vectors were extracted, and the final concentration reached 1 ug/uL for use. Appropriate amounts of calli were put into the culture dishes, and an enzymatic solution was added to separate the cell walls from the protoplasts. They were then placed in a shaker at 27 °C at 40 rpm and enzymolyzed in the dark for 3–6 h. The carrier plasmid was added to the isolated and purified protoplast solutions, and a PEG solution of equal volume was added. The vectors were transferred into protoplasts via PEG mediation. Finally, the positions of GFP and mCherry were observed and photographed with a confocal laser microscope.

In order to further verify the subcellular localization of LcCOBL proteins, onion epidermal cells were transformed with the 35S: *LcCOBL2, 4 5*-*GFP* vectors by gene gun, with *35S: GFP* vector as a control. The bombarded cells were incubated in the dark at 22–24 °C for 12–24 h to allow transient expression of the proteins. The GFP location was observed and photographed with a fluorescent microscope.

## 5. Conclusions

In this study, a genome-wide analysis of the *LcCOBL* gene family was conducted by focusing on their genetic structures and responses to stress treatments, and a total of seven *LcCOBL* genes were identified. The chromosomal distributions, gene structures and motifs, cis-regulatory elements in the promoter region, and expression patterns of the *LcCOBL* genes under different stress treatments and subcellular localizations were analyzed. The subcellular localization analysis and experimental results show that the *LcCOBL* genes are localized in the cytoplasm. The tissue expression pattern analysis of the *LcCOBL* genes shows that the *LCCOBL* genes are highly expressed in plant stems and may be involved in cellulose biosynthesis. The *LcCOBL* genes showed different response trends under cold, heat and drought stress treatments, indicating that they play a role in coping with environmental stresses. These results provide basic information on the *COBL* gene family and a good platform for exploring the specific roles of these genes in stress tolerance and the development of *L. chinense*.

## Figures and Tables

**Table 1:** List of gene IDs and characteristics of the *LcCOBL* genes.

Gene Name	Gene ID	Locus	MW (kDa) ^a^	pI ^b^	GRAVY ^c^	N-Terminal Signal Peptide	GPI Modification Site	Subcellular Location
*LcCOBL1*	Lchi01857	Chr2	42.96	8.43	−0.271	Yes	Yes	Cell membrane
*LcCOBL2*	Lchi02570	Chr5	59.66	8.81	−0.245	Yes	No	Cell membrane; chloroplasts
*LcCOBL3*	Lchi20746	Chr10	70.30	5.53	−0.059	Yes	Yes	Cell membrane
*LcCOBL4*	Lchi12311	Chr13	40.58	8.95	−0.199	Yes	No	Cell membrane
*LcCOBL5*	Lchi14677	Chr16	25.30	7.53	−0.239	Yes	No	Cell membrane
*LcCOBL6*	Lchi21892	Chr19	73.59	8.47	−0.28	Yes	Yes	Cell membrane
*LcCOBL7*	Lchi34460	Contig2982	73.66	8.45	−0.271	Yes	Yes	Cell membrane

^a^ Molecular weight. ^b^ Isoectric point. ^c^ Grand average of hydropathy.

## Data Availability

The original contributions presented in this study are publicly available. The *L. chinense* protein sequences can be found at https://hardwoodgenomics.org/Genome-assembly/2630420 (accessed on 3 March 2022). The RNA sequences can be obtained from the NCBI, accession numbers PRJNA679089 and PRJNA679101.

## Supplementary Materials

The following supporting information can be downloaded at https://www.mdpi.com/article/10.3390/plants12081616/s1. Figure S1: Hydrophobicity analysis of amino acid sequences of LcCOBLs; Figure S2: Prediction results of GPI modification sites of protein sequences of LcCOBL; Figure S3: Prediction results of signal peptides of protein sequences of LcCOBL; Figure S4: Prediction results of transmembrane domains of LcCOBL proteins; Figure S5: ClustalW was used for multi-sequence alignment of LcCOBL protein sequences; Figure S6: Amino acid sequences of each motif; Figure S7: Subcellular localization of LcCOBL2, 4 proteins in *L. chinense* calli and in onion epidermal cells; Table S1: Protein names and sequences of COBL; Table S2: Primer sequences of LcCOBL proteins for subcellular localization; Table S3: Primer sequences of *LcCOBLs* and reference genes in qRT-PCR.
