# Supplementary material for: Genome-Wide Analysis and Abiotic Stress-Responsive Patterns of COBRA-like Gene Family in Liriodendron chinense
Source: Plants (Basel). 2023 Apr 11;12(8):1616. doi: 10.3390/plants12081616 (PMC10143436; doi:10.3390/plants12081616)
Supplement: Supplementary file 1 [file plants-12-01616-s001.zip › Supplementary Figure.pdf]

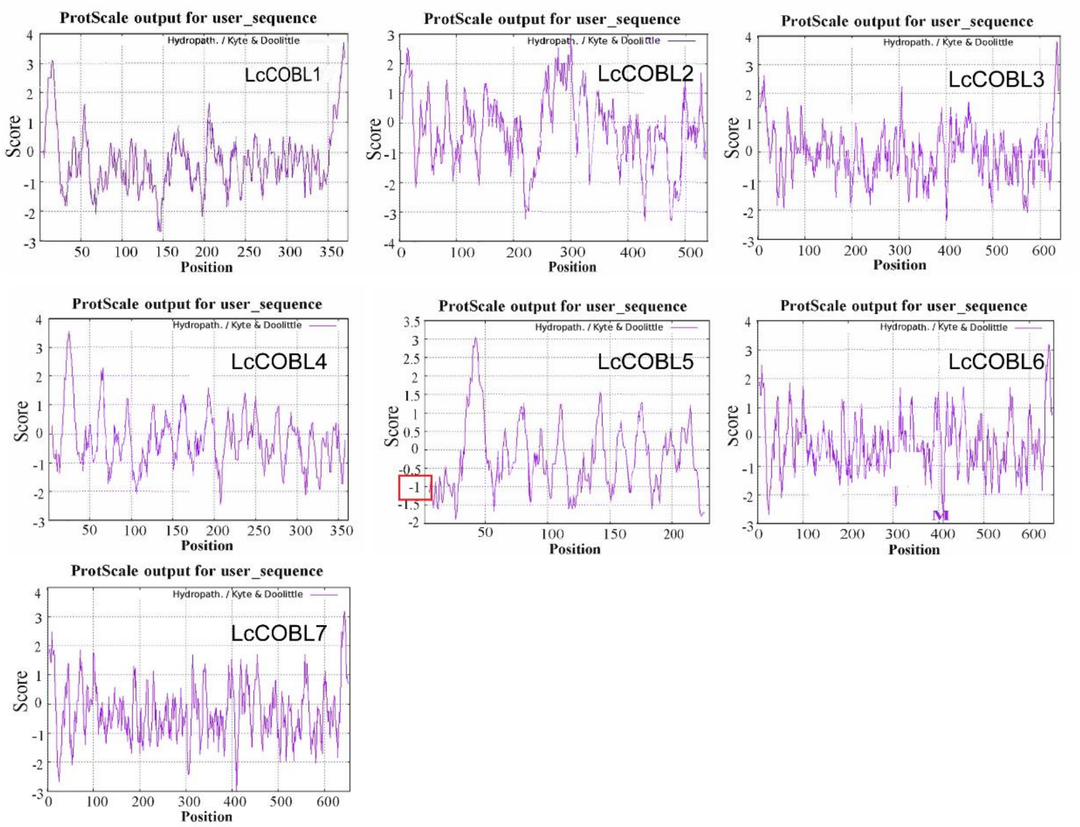

Supplementary Figure S1. Hydrophobicity analysis of amino acid sequences of LcCOBL. The vertical axis indicates hydrophilicity (positive value) or hydrophilicity (negative value). The horizontal axis represents the length of the amino acids.

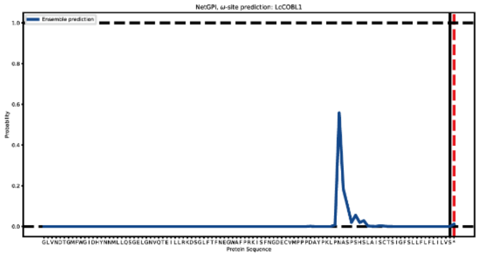

LcCOBL1

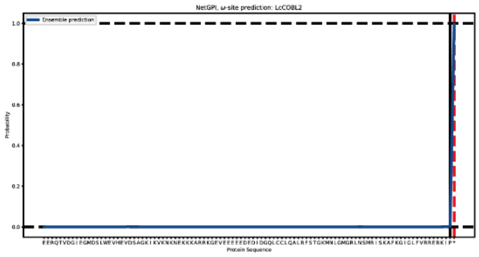

LcCOBL2

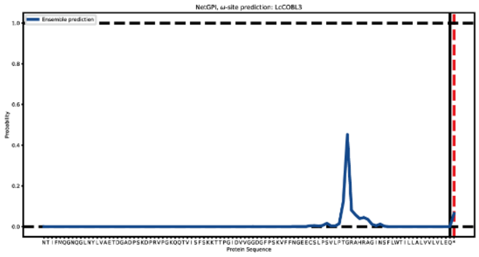

LcCOBL3

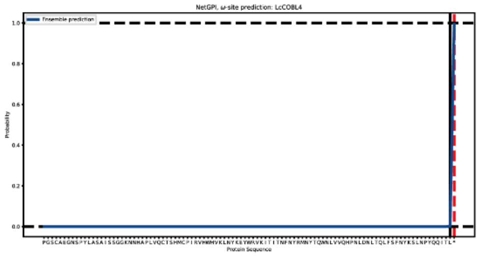

LcCOBL4

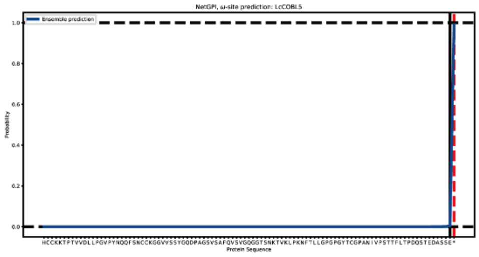

LcCOBL5

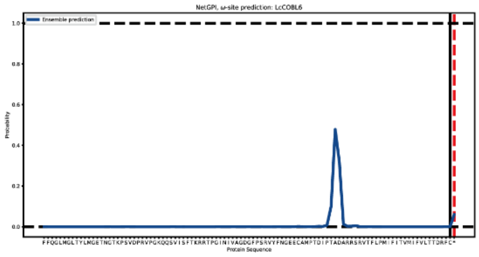

LcCOBL6

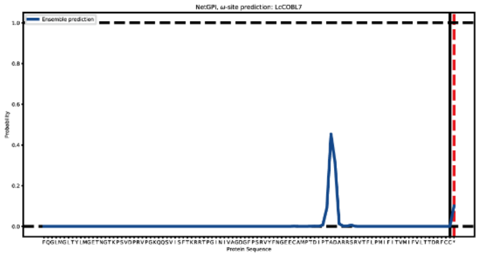

LcCOBL7

Supplementary Figure S2: Prediction results of GPI modification sites of protein sequences of LcCOBL.

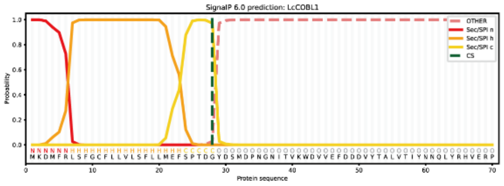

LcCOBL1

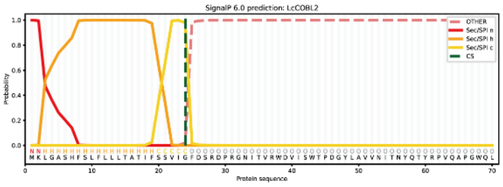

LcCOBL2

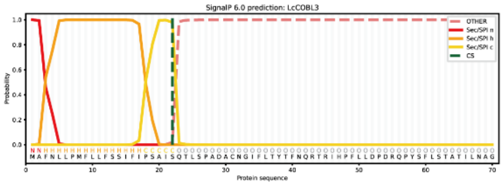

LcCOBL3

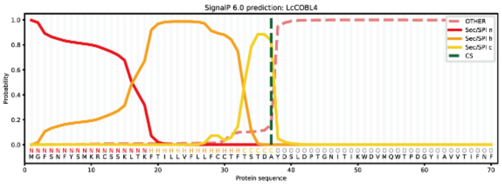

LcCOBL4

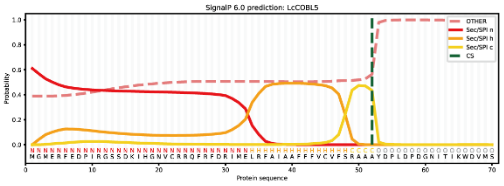

LcCOBL5

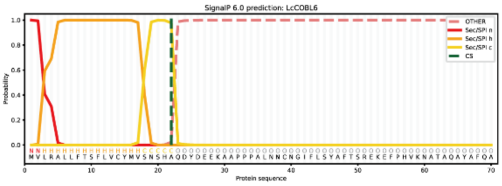

LcCOBL6

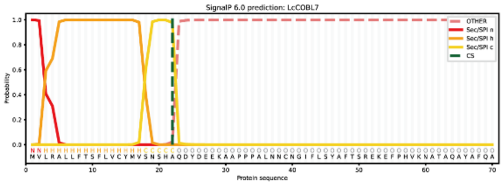

LcCOBL7

Supplementary Figure S3: Prediction results of signal peptides of protein sequences of LcCOBL.

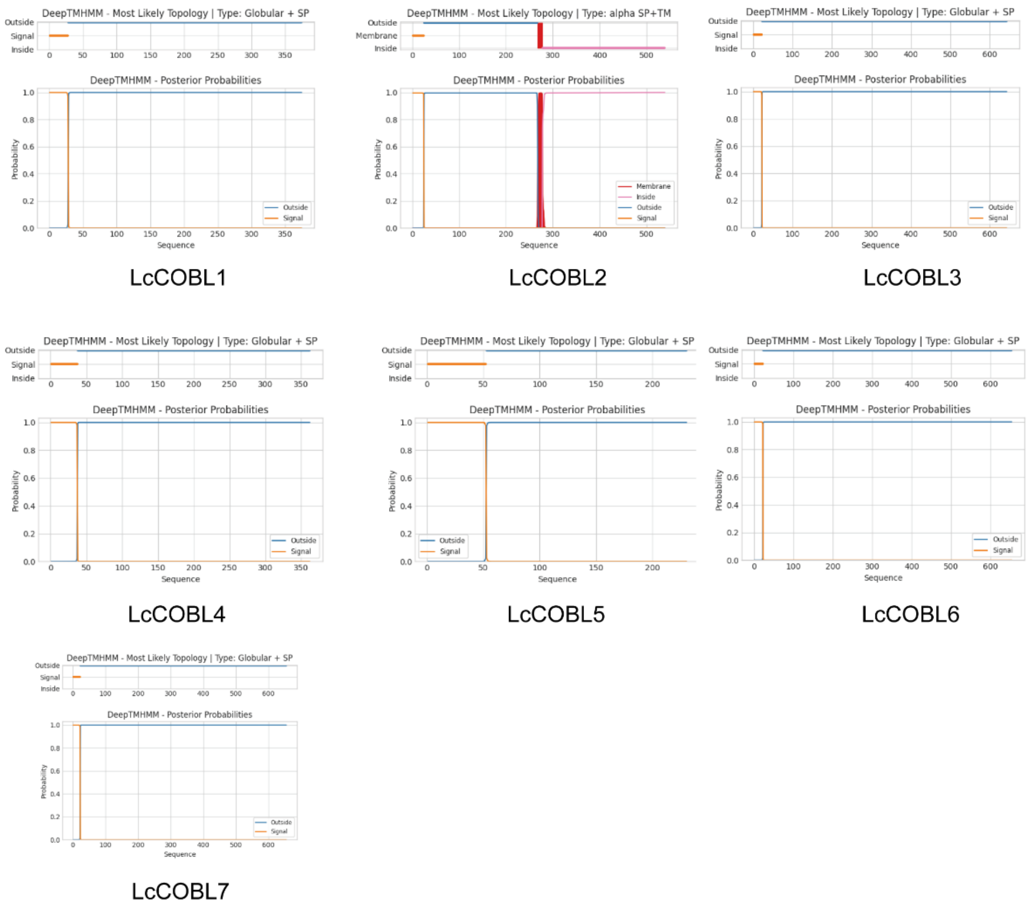

Supplementary Figure S4: Prediction results of transmembrane domains of LcCOBL proteins.

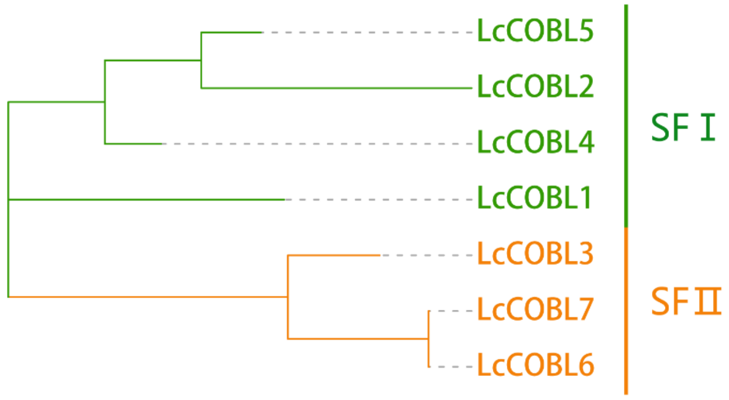

Supplementary Figure S5. ClustalW was used for multi-sequence alignment of LcCOBL protein sequences. The Maximum Likelihood (ML) tree was built using MEGA X and 1000 bootstrap repeats. The tree was visualized with the online iToL.

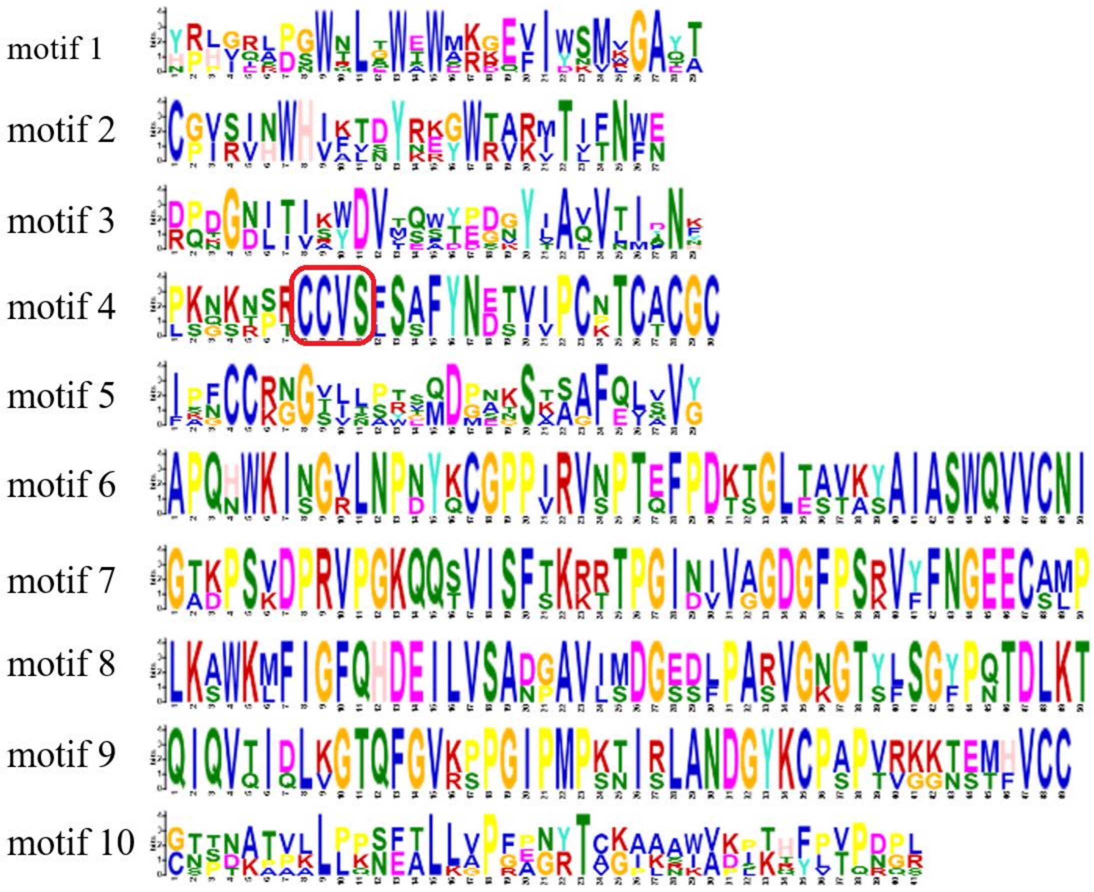

Supplementary Figure S6. Amino acid sequences of each motif. The font size represents the frequency of the respective amino acid.

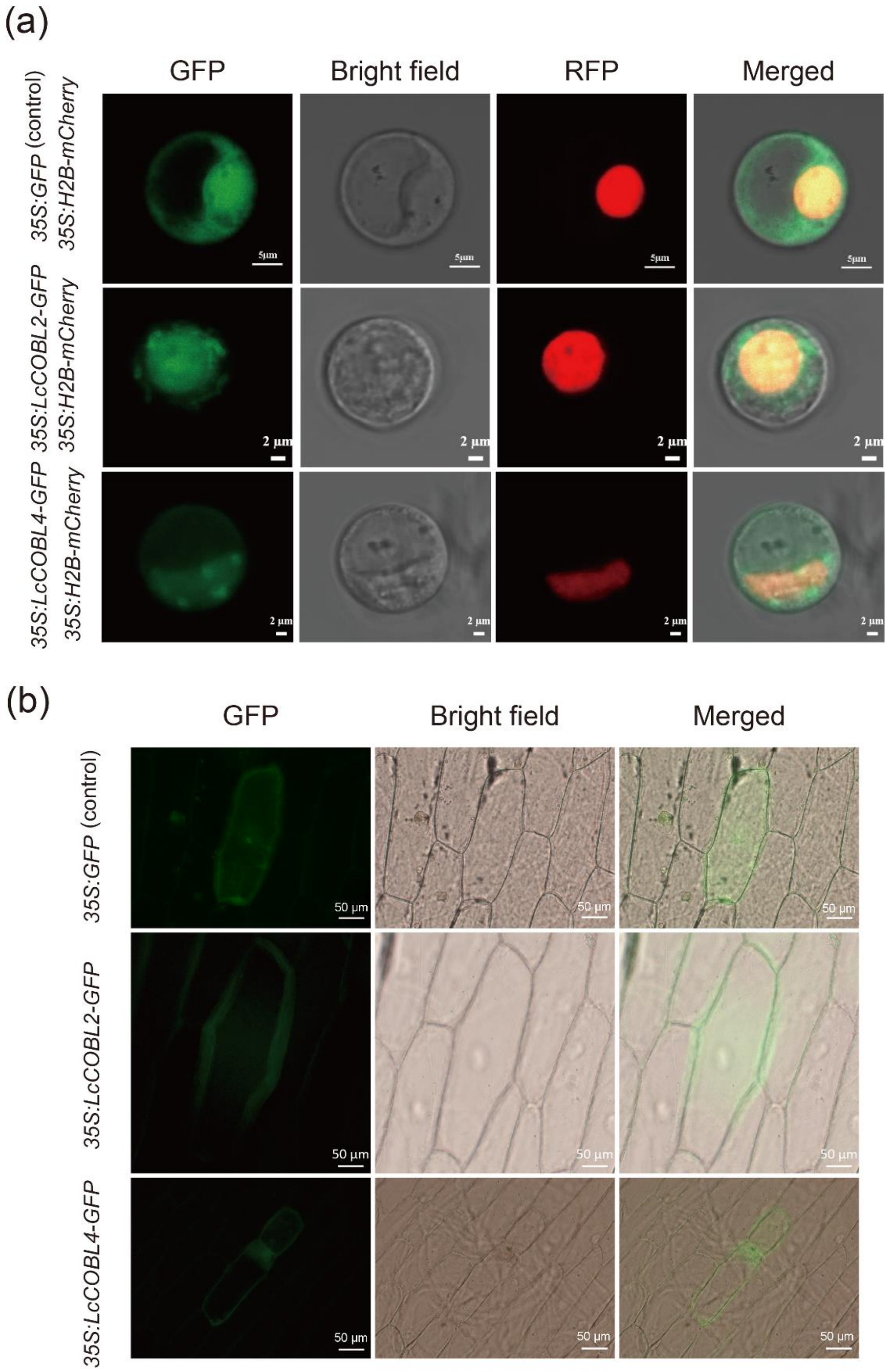

Supplementary Figure S7: Subcellular localization of LcCOBL2, 4 proteins in *L. chinense* calli (a) and in onion epidermal cells (b). In these pictures, GFP indicates green fluorescence photography, bright field indicates bright-field photography, RFP indicates red fluorescence photography, and merged indicates the fusion of green fluorescence, red f fluorescence and bright field photography. The red fluorescence locates the nucleus, and the green fluorescence locates the LcCOBL proteins.
