# Supplementary figures and images for: Genome-Wide Analysis and Abiotic Stress-Responsive Patterns of COBRA-like Gene Family in Liriodendron chinense
Source: Plants (Basel). 2023 Apr 11;12(8):1616. doi: 10.3390/plants12081616 (PMC10143436; doi:10.3390/plants12081616)

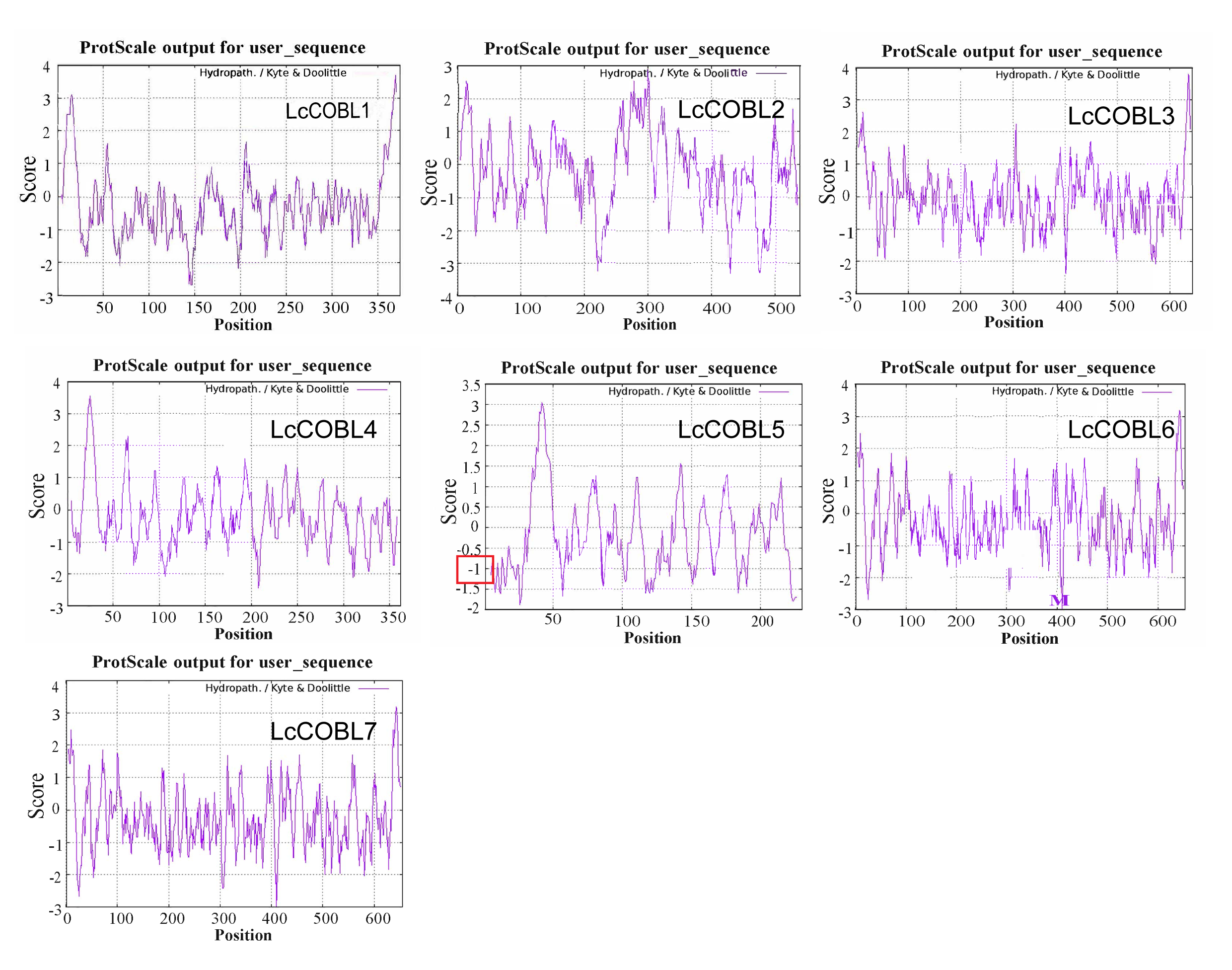

Supplement: Supplementary file 1 [file plants-12-01616-s001.zip › Figure S1.tif]

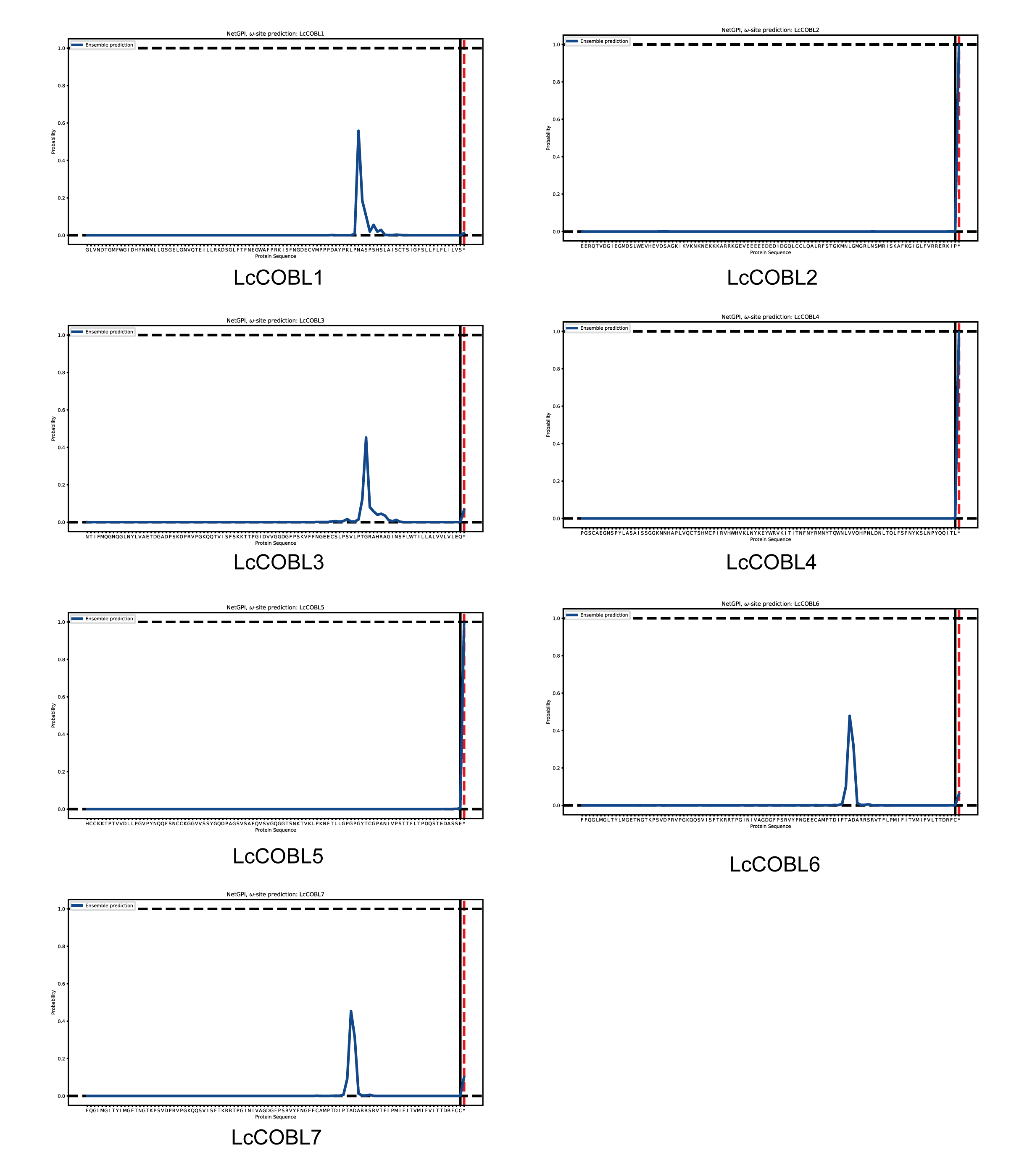

Supplement: Supplementary file 1 [file plants-12-01616-s001.zip › Figure S2.tif]

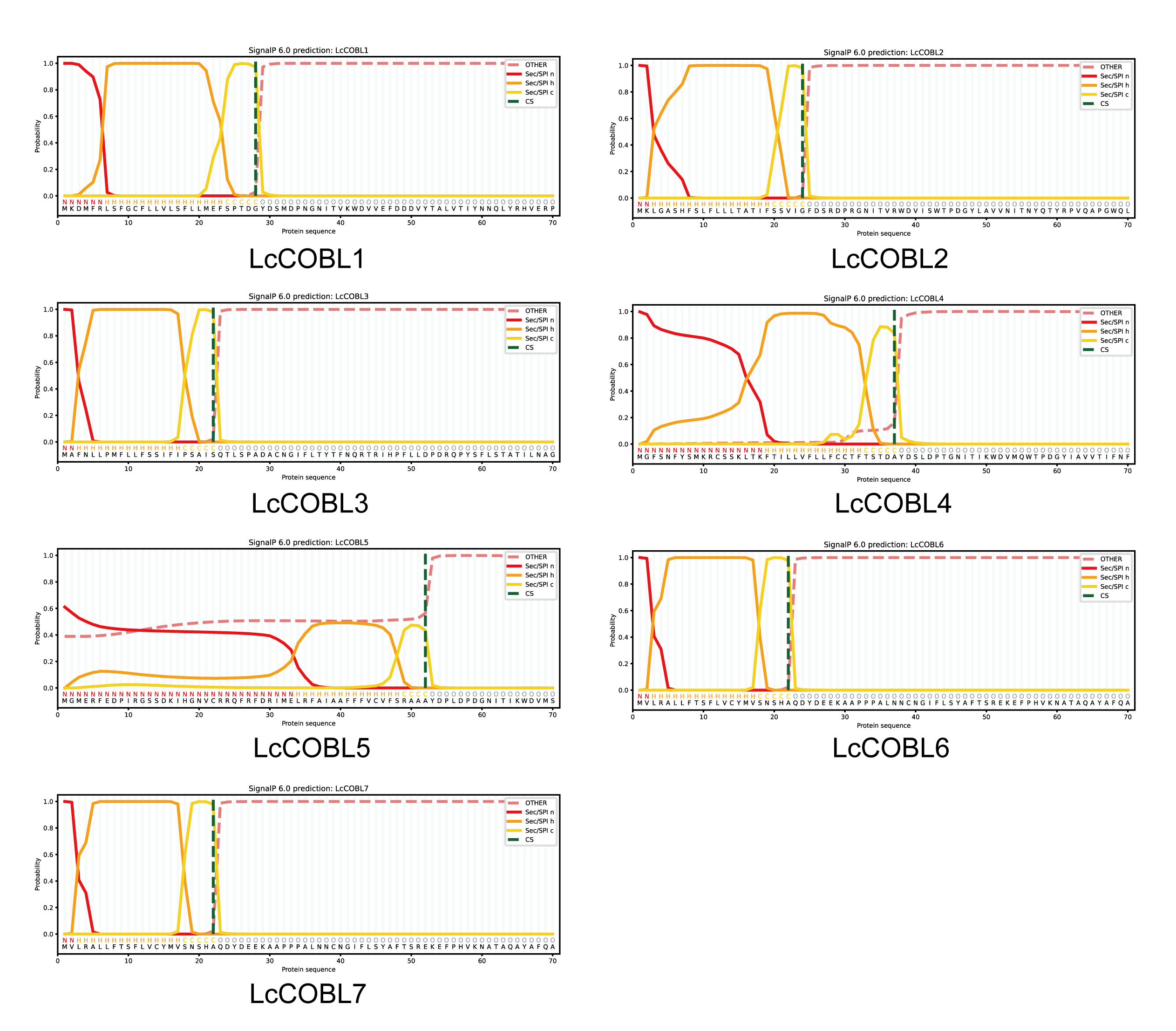

Supplement: Supplementary file 1 [file plants-12-01616-s001.zip › Figure S3.tif]

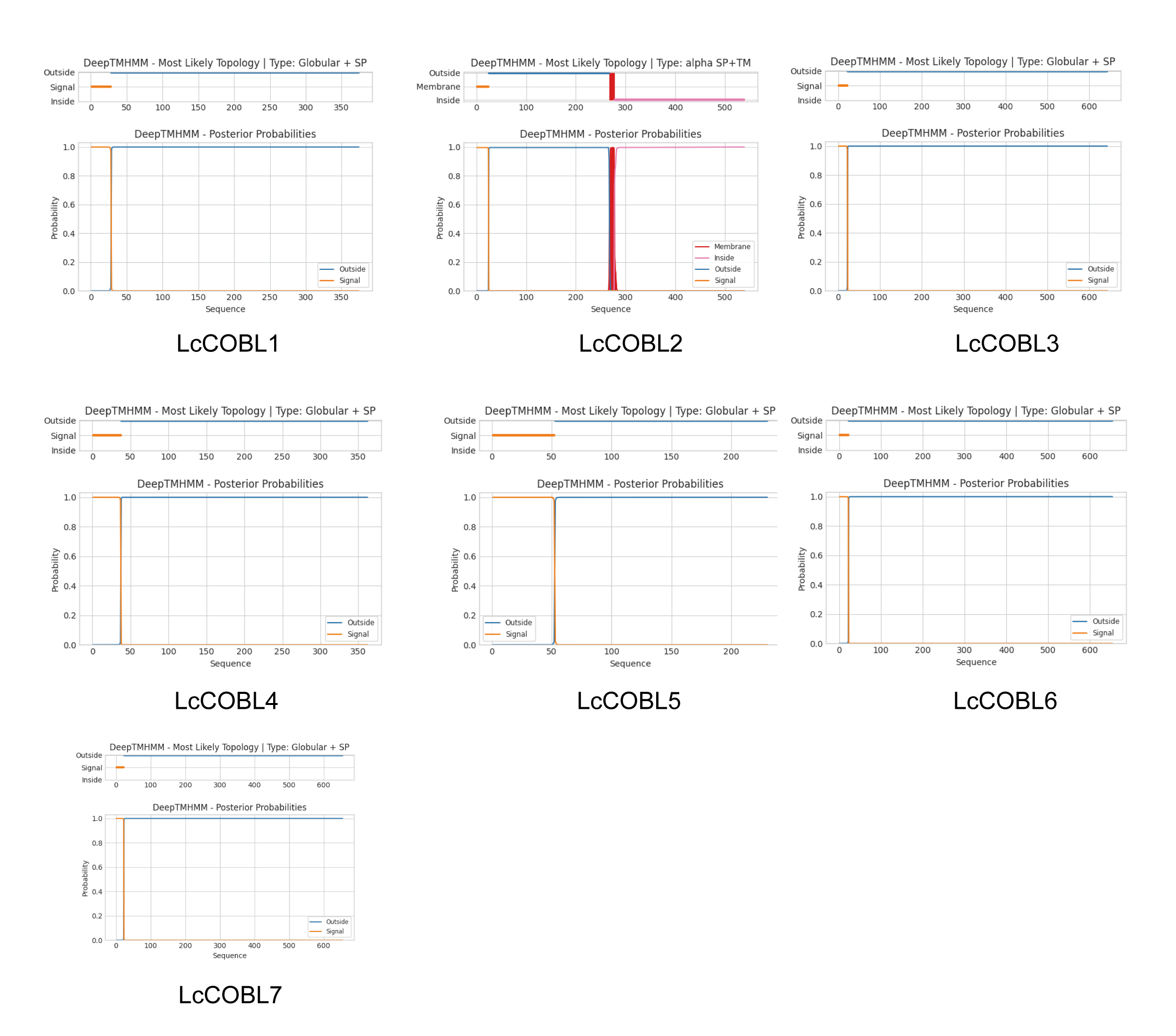

Supplement: Supplementary file 1 [file plants-12-01616-s001.zip › Figure S4.tif]

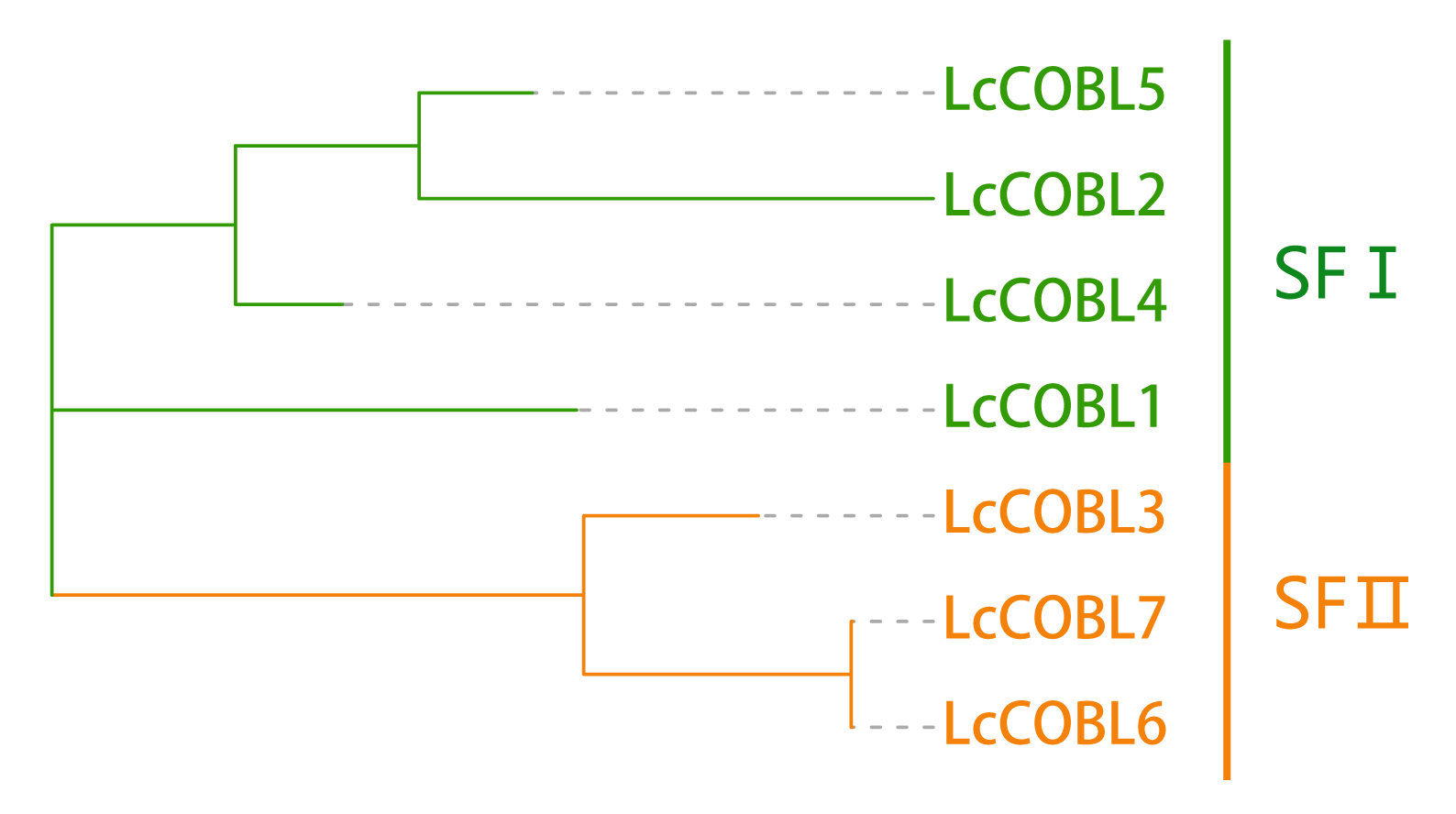

Supplement: Supplementary file 1 [file plants-12-01616-s001.zip › Figure S5.tif]

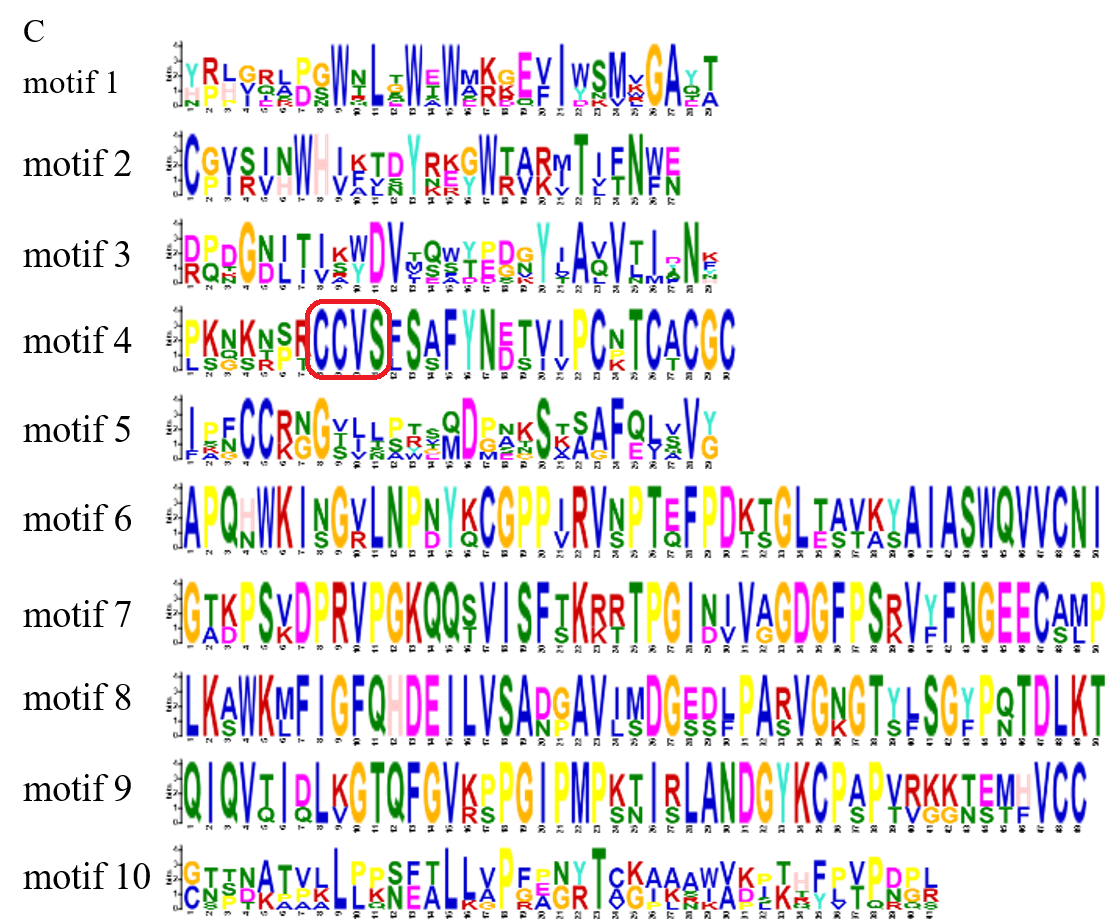

Supplement: Supplementary file 1 [file plants-12-01616-s001.zip › Figure S6.png]

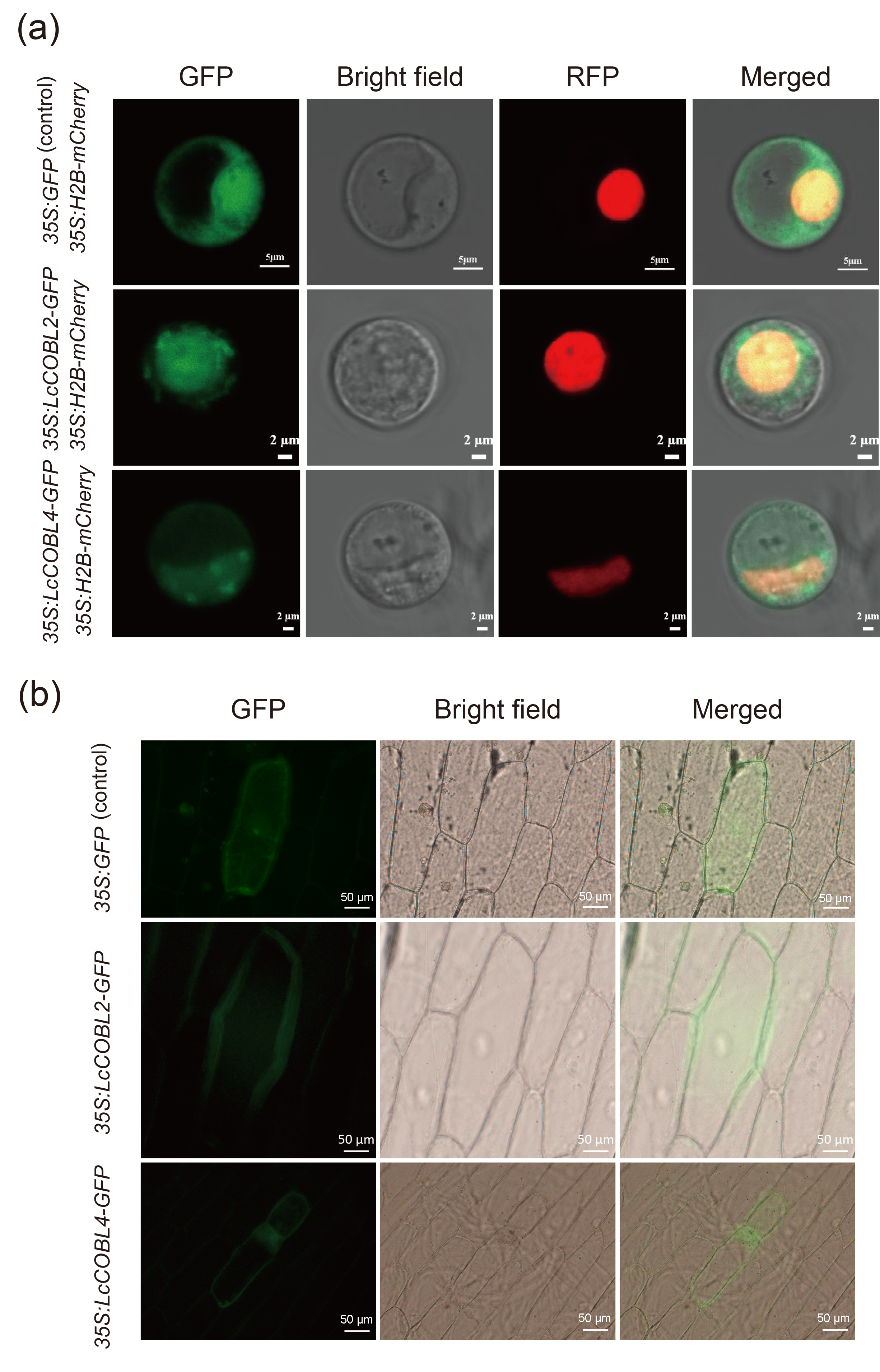

Supplement: Supplementary file 1 [file plants-12-01616-s001.zip › Figure S7.tif]
